# Clinical analysis of ultrasound‐guided microwave ablation for pediatric thyroid nodules‐ a single center research

**DOI:** 10.1002/pdi3.98

**Published:** 2024-07-01

**Authors:** Hongxia Zhang, Mingzhu Yu, Jingyu Chen, Caihui Hu, Yi Tang, Zhenzhen Zhao, Yifei Du, Jian Sun, Jianwu Zhou, Chao Yang, Xiaobin Deng, Xiangru Kong

**Affiliations:** ^1^ Department of Surgical Oncology Ministry of Education Key Laboratory of Child Development and Disorders Chongqing Key Laboratory of Pediatrics Children's Hospital of Chongqing Medical University National Clinical Research Center for Child Health and Disorders Chongqing China; ^2^ Department of Ultrasound Children's Hospital of Chongqing Medical University Chongqing China

**Keywords:** benign thyroid nodules, children, microwave ablation, thyroid hormones

## Abstract

To provide a new minimally invasive treatment for children with benign thyroid nodules, and to provide clinical data for applying microwave ablation (MWA) to children. A retrospective analysis was conducted on the clinical data of 21 pediatric patients with benign thyroid nodules who underwent ultrasound‐guided MWA at the Children's Hospital affiliated with Chongqing Medical University from July 2022 to August 2023. The safety, clinical efficacy, volume reduction ratio and prognostic value of the treatment were evaluated. The participants were followed for at least 4 months (median 7 months, range 4–16 months). The average (range) ablation time for the 21 patients was only(233.90 ± 184.97) (40–660)seconds, with intraoperative bleeding less than 0.5 mL. No complications such as hoarseness, seizures or coughing during drinking water were observed after ablation treatment. All the participants' hormone reflecting thyroid function remained in the normal ranges after treatment. Besides, these hormones at 12 h after surgery and 1 month after surgery were not statistically different from those before surgery. Immediate postoperative ultrasound imaging showed a significant decrease in volume of benign thyroid nodules, the volume of nodules at 1 month postoperatively (M, 1.39), and the volume of nodules at 4 months postoperatively (M, 0.40) significantly smaller than that before surgery (M, 4.94). Ultrasound‐guided MWA is a new option for the treatment of benign thyroid nodules in children, with advantages such as minimal invasiveness, good clinical effect, high safety, little damage to thyroid function, short operation time, less intraoperative bleeding, low pain sensation and aesthetic appearance.

## Graphical abstract

Microwave ablation (MWA) is a minimally invasive treatment method for pediatric thyroid nodules. Our study aimed to assess the efficacy and safety of MWA in reducing the size of these nodules. MWA utilizes microwave energy to destroy abnormal thyroid tissue, resulting in nodule shrinkage and symptom relief. Our findings demonstrate significant volume reduction and high safety of MWA treatment. It effectively reduces nodules, alleviates symptoms of airway and esophageal compression, and preserves thyroid function. MWA offers a minimally invasive approach, minimizing hospitalization and recovery time. Our study concludes that MWA is a promising treatment option for pediatric thyroid nodules, providing a safe and effective choice for affected children. Further research is required to evaluate long‐term outcomes and compare it with other treatment modalities.

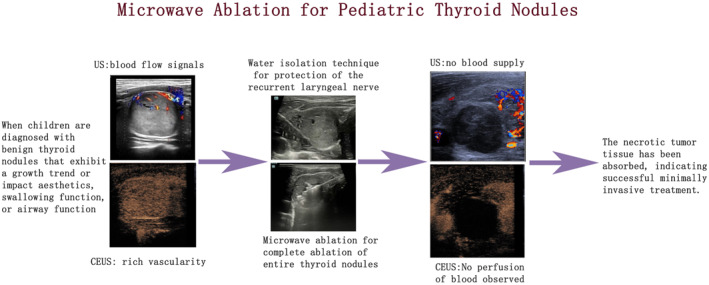

## 1 INTRODUCTION

Thyroid nodules are quite common in clinics. Most nodules are harmless, but they still make patients feel unhappy and uneasy.^1^ The incidence rate of thyroid nodules in children ranges from 0.2% to 5%, which is lower than that in adult patients.^2^ A recent population‐based study of Japanese adolescents found that more than 50% of school‐age children have thyroid cysts.^3^ In addition, they may develop into tumors if they are not controlled. Pediatric thyroid nodules have a higher malignancy rate of 22%–26% compared to adult thyroid nodules.^4^, ^5^, ^6^ The malignancy rate is especially high for single nodules, and it increases with nodule size.^7^

The thyroid gland plays an important role in the growth and development of children. Improper treatment can aggravate the condition, lead to various clinical symptoms, such as respiratory, or swallowing function limitation, and may also lead to appearance changes.^8^ Therefore, timely treatment of thyroid nodules is essential. Currently, surgical resection is the primary treatment method for thyroid nodules in children. However, there are drawbacks to surgery, such as a 2%–10% complication rate, high incidence of hypoparathyroidism, scar formation,^9^ and destruction of normal thyroid tissue. These drawbacks may potentially cause long‐term health damage to the child.^8^ Therefore, increasing nonsurgical and minimally invasive treatments, such as microwave ablation (MWA), percutaneous ethanol injection, to laser ablation and radiofrequency ablation (RFA), have been developed rapidly in recent years to treat this disease. Numerous studies have been conducted to investigate the safety and efficacy of RFA for treating benign tumors in pediatric patients with thyroid nodules.^10^, ^11^, ^12^ However, there is a problem with a high regeneration rate (about 22.9%–24%) when treating thyroid nodules with RFA.^11^ MWA has several advantages over RFA, including better heating of larger tumors and less heat sink effect, because MWA is faster and more effective.^13^

Most recently, MWA has been advocated to treat thyroid nodules, drawing on knowledge from its usage in other organs, particularly the liver,^14^ kidney,^15^ and lung.^16^ It is a pity that there is only limited relevant clinical research focusing on MWA in pediatric patients due to the late application of interventional ultrasound technology in children.^17^ Therefore, we designed this study to explore the safety and effectiveness of MWA for thyroid nodules in children.

## 2 METHODS

### 2.1 Materials and methods

#### 2.1.1 Clinical information

**FIGURE 1:**
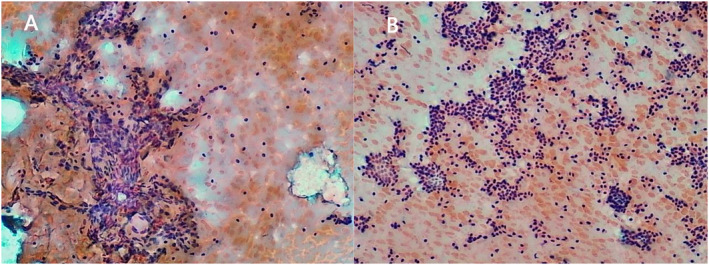
A&B are pathological pictures of a 9‐year‐old girl with mixed thyroid nodules.

Twenty one pediatric patients, who were diagnosed with benign thyroid nodules at the Children's Hospital Affiliated to Chongqing Medical University from July 2022 to August 2023, were included in this study. All patients underwent a percutaneous MWA guided by ultrasound for treatment. A total of 34 nodules were ablated. The nodules were divided in three types according to its compositions: mainly solid (>80% solid), mainly cystic (>80% liquid) or mixed type. Finally, 14 cases of adenoma, and 7 cases of cystadenoma were confirmed by pathology (Figure 1). All patients voluntarily participated in this study and signed an informed consent form. The ethics committee of our hospital was fully informed about this study and approved the research (Ethics Approval Number: Chongqing Medical University Children's Hospital Approval Notice 009.2009).

### 2.2 Inclusion criteria and exclusion criteria

#### 2.2.1 Inclusion criteria

①Thyroid nodules were diagnosed as benign through needle biopsy and pathological evaluation; ②Patients demonstrated with self‐perceived symptoms related to the nodules (such as foreign body sensation, discomfort in the neck, etc.), or had aesthetic concerns for requesting treatment; ③Informed consent was obtained from the legal guardian of the pediatric patient to undergo percutaneous MWA under ultrasound guidance; ④Patients showed no contraindications for surgery; ⑤It is the initial diagnosis for patients with no prior treatment performed.

#### 2.2.2 Exclusion criteria

①Patients had other thyroid diseases concurrently; ②Patients underwent a thyroid surgery previously; ③Patients has dysfunction of the contralateral vocal cord; ④Patients had severe medical conditions; ⑤There are some surgical contraindications for patients.

### 2.3 Ablation methods

**FIGURE 2:**
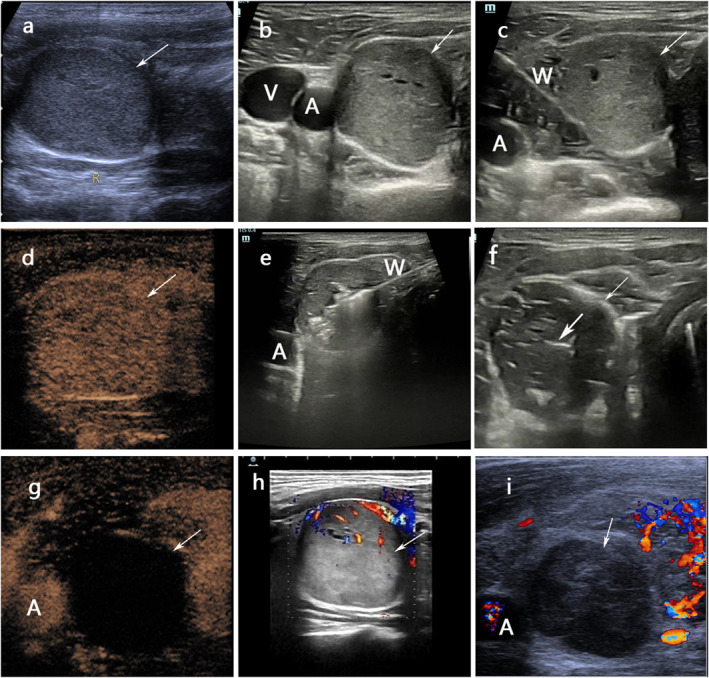
A 11‐year‐old boy received microwave ablation (MWA) for a solid benign nodule in the right lobe of his thyroid gland. (A) Longitudinal image before MWA showed a isoechoic solid nodule (thick arrow) with peripheral halo (the nodules were measured as4.0 cm × 2.44 cm × 2.03 cm). (B) Transverse images before treatment showed the nodules adjacent carotid artery(A). (C). Saline(W) is injected separately into the carotid sheath, thyroid lateral, and posterior spaces to form a water barrier to protect the recurrent laryngeal nerve and blood vessel. (D) CEUS before treatment displays hyperenhancement of the nodule. (E) Microwave antenna (M) was positioned in the nodule, “moving‐shot” technology was used until the nodule was covered by the hyperechoic microbubbles. (F) There were necrotic tissue (the thick arrow) in the nodules after MWA. (G) CEUS after treatment, no contrast agent was presented in the nodule. (H) Color Doppler showed intra‐nodular with vascularity before MWA. (I) Color Doppler shows intra‐nodular without vascularity.

All patients who were under general anesthesia, in a supine position and the chin raised to fully expose the thyroid gland, were ablated under ultrasound (Mindray M10 Portable Ultrasound Machine) guidance. Firstly, under ultrasound guidance, a trans‐isthmic approach was generally used, and saline is injected separately into the thyroid and the trachea, esophagus, carotid sheath, thyroid lateral, and posterior spaces to form a water barrier to protect the recurrent laryngeal nerve and esophagus (Figure 2C). Then, we used Nanjing Kangyou Microwave Ablation System to ablation, and the microwave puncture needle was inserted into the l nodules, following the "from far to near" or "from deep to shallow" principle,^18^ and ablation is performed at a power of 25–30W. Fixed ablation is used for smaller nodules, while moving ablation is used for larger nodules (Figure 2E). Besides, for lesions with a large cystic change range, liquid is firstly aspirated before ablation. During the procedure, Contrast‐enhanced ultrasound (CEUS) (Sono Vue R used as a contrast agent) was performed to determine whether the targeted nodules had been completely ablated, and the supplement ablation was used in areas with incomplete ablation (Figure 2D,G). After the surgery, the incision was covered with a dressing and continuous pressure was applied for 15 min to prevent bleeding or hematoma formation. Figure 2A represents the longitudinal section of a thyroid nodule, while Figure 2B shows its transverse section and depicts arterial and venous relationships with the nodule. Figure 2F provides an overview of a nodule after ablation, and Figure 2H and I display blood flow patterns before and after ablation respectively.

### 2.4 Observation indexes

All patients underwent blood examination before surgery, at 12 h after surgery, and 1 month after surgery. The levels of serum thyroid‐stimulating hormone (TSH), free triiodothyronine (FT3), and free thyroxine (FT4) were measured using the immunoradiometric assay method. Postoperatively, the Wong‐Baker Pain Rating Scale (FACES) was used to assess postoperative pain in patients, and any complications such as choking water, hoarseness, local swelling, bleeding, or seizures were recorded in detail. Besides，the operation time and intraoperative blood loss were recorded.

All patients underwent regular ultrasound or contrast‐enhanced ultrasound examinations before surgery, 1 month after surgery, and 4 months after surgery. Patients underwent ultrasound combined with fine needle aspiration biopsy for definitive diagnosis. The following formulas were used to evaluate the efficacy: The nodule volume was calculated using the equation: *V* = 0.525× (a× b× c) (where V is the volume, a is the maximum diameter, b and c are the other two perpendicular diameters)^19^; Volume Reduction Rate [Volume reduction ratio (VRR) (%)] = (preoperative volume‐postoperative volume)/preoperative volume×100%.

### 2.5 Statistical methods

Statistical analysis was conducted using SPSS 23.0. Normally distributed continuous data were expressed as the mean (M) with 95% CI and a skewed distribution data were reported as the median and range. Continuous datasets were compared using the paired *t*‐test or one‐way analysis of variance, and non‐normally distributed data were analyzed using the Mann–Whitney *U* test. *p* < 0.05 indicates statistically significant differences.

## 3 RESULTS

### 3.1 General information of patients and thyroid nodules

A total of 21 pediatric patients were included in this study, with 5 (24%) males and 16 (76%) females. The average (range) age of the patients was 9.53 (5.25–15.67) years old. There were 34 nodules in the 21 patients, including 14 nodules in 14 cases (67%) with a single nodule and 20 nodules in 7 cases (33%) with multiple nodules. The type of nodules was mainly mixed type (solid and cystic, 22 [65%] nodules) and solid type (12 [35%] nodules). The mean power of ablation was 25W, and the mean (range) ablation time was 233.90 (40–660) seconds. The mean (range) diameter of the largest nodule was 1.93 (0.3–5.7) cm. Prior to the minimally invasive MWA surgery, the mean (range) volume of the nodules was 4.94 (0.08–31.06) cm³. Please refer to Table 1 for more details.

**TABLE 1:** The clinical features of patients and nodules.

Variable	Datum
No. of patients	21
Mean age (years)^a^	(9.53 ± 2.60) (5.25–15.67)
Gender(Female/Male)	16(76%)/5(24%)
No. of nodules	34
Mainly solid nodules/Mixed nodules	12(35%)/22(65%)
A single nodule/Multiple nodules	14(67%)/7(33%)
Mean nodules diameters^a^, cm	(1.93 ± 1.19) (0.3–5.7)
Mean nodules volumes^a^, (cm)	(4.94 ± 6.50) (0.08–31.06).
Locations of nodules (Left/right/isthmus)	19(56%)/15(44%)/‐
Mean ablation time^a^, seconds	(233.90 ± 184.97) (40–660)

^a^ Data are means ± standard deviation; data in parentheses are ranges or percentages.

### 3.2 Surgical indicators of ultrasound‐guided MWA

#### 3.2.1 High operative success rate of MWA

Among the 34 thyroid nodules in the 21 patients, immediate postoperative ultrasound examination showed no contrast agent perfusion within the original nodule location, indicating the complete ablation for all nodules in our minimal invasive surgery of ultrasound‐guided MWA.

### 3.3 Less surgical duration and less intraoperative bleeding

Among the 21 cases, the surgical duration ranged from 40 to 660 s (M, 233.90), and the amount of bleeding was between 0.1 and 0.5 mL.

### 3.4 Aesthetically pleasing

The needle incision in the neck was only 1 mm, with no redness or swelling observed in the skin around the incision. Both the family members and patients were highly satisfied with the less wound induced by the minimal invasive surgery of ultrasound‐guided MWA.

### 3.5 Changes in volume of thyroid nodules

**FIGURE 3:**
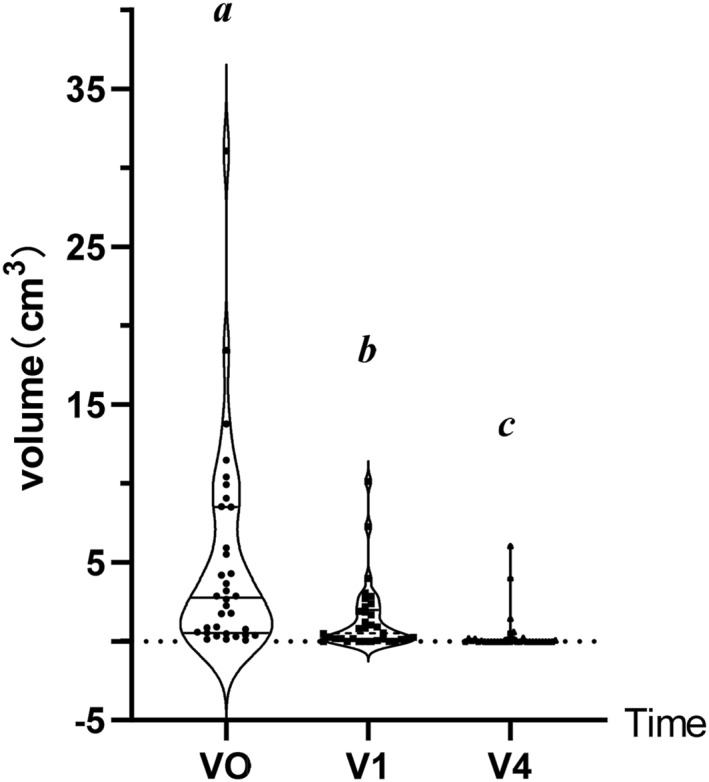
Changes in total thyroid nodule average volume.

The volume of thyroid nodules was examined by the conventional ultrasonography (Siemens ACUSON Sequoia Ultrasound Machine). Before the minimal invasive surgery, the mean (M) size of thyroid nodules was 4.94 (95% CI, 2.67–7.20) cm^3^, including mixed type (M, 5.93; 95% CI, 2.67–9.19) and solid type (M, 3.11; 95% CI, 0.43–5.80). However, at 1 month after the minimal invasive surgery, the volume of benign thyroid nodules decreased significantly (M, 1.39; 95% CI, 0.63–2.50), including mixed type(M, 1.48; 95% CI, 0.48–2.49; *p* > 0.05) and solid type(M, 1.21; 95% CI, −0.10–2.54; *p* > 0.05). At 4 months after the minimal invasive surgery of MWA, the volume of residual benign thyroid nodule (M, 0.40; 95% CI, −0.03–0.82) was smaller than that of pre‐operation. The mean volume of mixed type and solid type were 0.31 cm^3^ (95% CI, −0.09–0.70) and 0.56 cm^3^ (95% CI, −0.56–1.67) separately. The VRR of post‐treatment at 1 month and 4 months were 70% and 94%, respectively (Table 2 and Figure 3).

**TABLE 2:** Changes in volume of thyroid nodules.

Item	Type	Before MWA	1 month after MWA	4 months after MWA
Volume(cm^3^) (95% CI)	Mixed type	5.93(2.67–9.19)	1.48(0.48–2.49)	0.31(−0.09–0.70)
	Solid type	3.11(0.43–5.80)	1.21(−0.10–2.54)	0.56(−0.56–1.67)
	Total thyroid nodule	4.94(2.67–7.20)	1.39(0.63–2.50)	0.40(−0.03–0.82)
Average VRR (%)	Mixed type		73.84	94.92
	Solid type		40.17	93.73
	Total thyroid nodule		70	94

*Note*: * indicates comparison with the time before MWA.

### 3.6 The influence of thyroid function

The serum FT3 level at 12 h postoperatively (M, 7.99; 95% CI, 7.06–8.93; *p* = 0.29) and at 1 month postoperatively (M, 8.16; 95% CI, 7.59–8.72; *p* = 0.17) was no significantly difference compared to preoperative levels (M, 7.43; 95% CI, 6.67–8.19).The serum FT4 level at 12 h postoperatively (M, 21.06; 95% CI, 13.03–29.09; *p* = 0.43) and at 1 month postoperatively (M, 17.33; 95% CI, 14.76–19.90; *p* = 0.54) was no significantly difference compared to preoperative levels (M, 15.50; 95% CI, 13.76–17.25).

**FIGURE 4:**
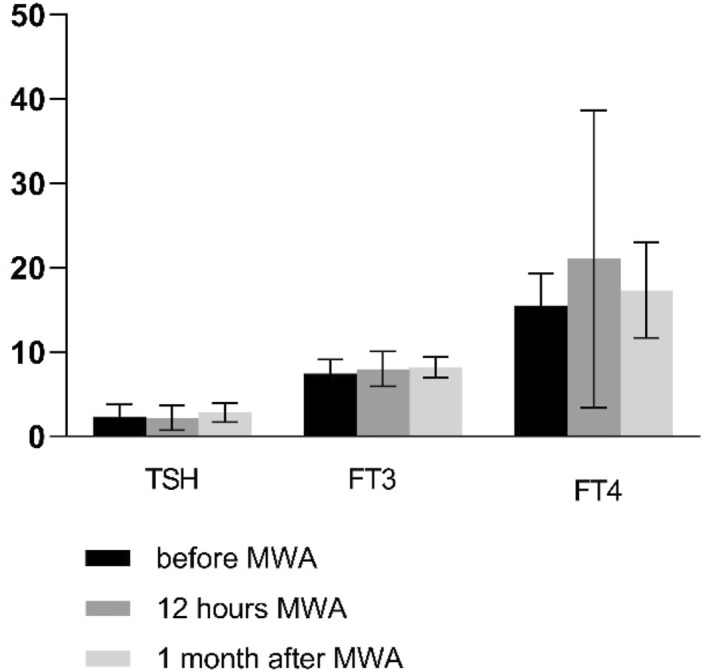
Changes in thyroid hormone.

There was no significant difference in serum TSH levels at 12 h postoperatively (M, 2.21; 95% CI, 1.53–2.88; *p* = 0.74) P mol/L compared to preoperative levels (M, 2.35; 95% CI, 2.70–1.99). The difference in TSH levels at 1 month postoperatively (M,2.82; 95% CI,2.30–2.34; *p* = 0.26) compared to preoperative levels (M, 2.35; 95% CI, 2.70–1.99) was not statistically significant difference (Figure 4).

### 3.7 Postoperative complications

Pain in the patients was assessed using the FACES. Within 12 h after surgery, the pain scores ranged from 2–4, but no pain was reported 24 h postoperatively, with scores ranging from 0 to 2. No complications such as hoarseness, convulsions, or coughing while drinking water occurred.

## 4 DISCUSSION

In this study, we evaluated the efficacy and safety of MWA in the treatment of children with thyroid nodules, and treated 21 children with benign thyroid nodules using MWA, with a total of 34 nodules ablated. CEUS was performed both preoperatively and immediately after surgery. The results confirmed that all nodules were successfully ablated. VRR was as high as 94% by the time of the final follow‐up assessment, confirming that MWA was effective in reducing the volume of nodules. In addition, during the postoperative follow‐ups, the thyroid hormones of the children were within the normal range and there were no postoperative complications, which confirmed that MWA was safe and reliable in children. Therefore, MWA has the advantages of minimal invasion, significant reduction of symptoms, and a high level of safety.

The total thyroid volume in children is smaller than that in adults. Thus, the nodules of the same size account for a larger proportion of normal thyroid tissue in children. Therefore, the pressure symptom of surrounding neck tissues is even more evident. Moreover, the nodules even exceed the volume of normal thyroid tissue in some children. Comparatively speaking, the blood vessels and nerves in the neck of children are denser. In addition, ablation therapy in adults is usually performed under the local anesthesia. The intraoperative conversations may quickly and effectively determine whether the recurrent laryngeal nerve is damaged or not. However, MWA shall be carried out under the general anesthesia in children to avoid the risks associated with non‐cooperation and accidental injury. Therefore, there is no way to determine whether the recurrent laryngeal nerve is injured by intraoperative talks. As a result, ablation procedures are more challenging in children with thyroid nodules than in adults. In summary, the treatment of thyroid nodules in children requires a safer and more precise technique. In contrast, conventional thyroidectomy has a higher incidence of complications such as intraoperative recurrent laryngeal nerve injury, postoperative hypoparathyroidism, and hypothyroidism.^20^, ^21^, ^22^, ^23^ Whereas MWA is performed under real‐time ultrasound monitoring and the local ablation range is accurate, which may achieve the maximum preservation of thyroid tissue on the basis of eliminating nodules. Besides, precise and effective water separation may increase the distance between the recurrent laryngeal nerve and the thyroid gland as well as remove some heat during the RFA, thereby partially avoiding damage to the recurrent laryngeal nerve.^24^, ^25^ In consequence, MWA effectively avoids the occurrence of these complications. In this study, 21 children with thyroid nodules achieved the complete ablation shown by CEUS immediately after MWA and there were no complications during the postoperative follow‐ups, indicating that the ablation effect of thyroid nodules was good in children. It is a new treatment method that can replace the traditional excision.

However, there are few studies on the application of thermal ablation to children with thyroid nodules. Moreover, the existing studies mainly focus on the RFA, while MWA has only one case reported. Shi et al.^26^ evaluated the safety and efficacy of MWA in 25 children (mean age of 9.84 ± 3.31 years, between 2 and 15 years old) with benign thyroid nodules. After a median follow‐up period of 12 months (6–48 months), the VRR was 85.0% at 1 year after operation, the children's subjective and objective symptoms were also improved, and the thyroid function was not impaired. The overall incidence of complications was 8.0% (2/25), which was bleeding and improved after treatment. No other complications or side effects were discovered. The recurrence rate of nodules was 16% (4/25). Li et al.^11^treated 70 nonfunctional benign thyroid nodules in 62 children using RFA, with a mean follow‐up period of 59.1 ± 10.5 months (48–85 months), a VRR of 77.5% at 1 year after operation but decreased to 55.1% at 4 years after operation. After treatment, the symptoms and appearance scores decreased significantly, but the incidence of nodule regrowth was 22.9%, and the overall incidence of complications was 4.8%. In this study, the VRR was as high as 94% at 4 months after surgery. Furthermore, after extended ablation, the nodules did not regrow or recur. All of them were ablated once. There were no complications after surgery. The VRR at 4 months after operation in the present study was higher than that in the study by Shi et al.^26^ and Li et al.11 However, the VRR was only 70% at 1 month after surgery. This is consistent with the previous findings of Ya Zhang et al.^27^ and Ren Yun et al.^28^ The reason may be that the extended ablation (i.e., 0.1–0.3 cm extended ablation outward on the basis of the original size of the nodule) is performed for the nodules in this study. The blood vessels supplying the nodules are often located at the edge of the nodules.^7^Therefore, expanded ablation allows for more complete ablation. At the same time, the nodule, however, appears larger on ultrasound than it actually is. Moreover, it takes time for the tumor to be absorbed after ablation, and the necrotic tissue is not completely absorbed in a short period of 1 month after surgery, but the overall ablation effect is better in the end. In addition, the postoperative recurrence rate in this study and the study of Shi et al.^26^ was significantly lower than that in the study of Li et al.^11^ The probable reason is that the MWA is more efficient and complete than the RFA. In the next place, the intraoperative use of CEUS may help to rapidly detect the presence of residual nodules and minimize them through timely supplemental ablation or avoid the recurrence resulting from incomplete tumor ablation.^7^In our study, it was found that the effects of solid nodules and mixed nodules were consistent 4 months after microwave ablation, and the volume of nodules was significantly smaller than that before ablation（*p* < 0.05）, which proved that microwave ablation had good tumor reduction effect on both types of nodules.

The efficacy and safety of MWA have been demonstrated in adults.^29^, ^30^, ^31^, ^32^ Du et al.^7^ performed the MWA under the local anesthesia in 148 adult patients (a mean age of 41.2 ± 10.7 years, between 25 and 72 years old) with benign thyroid nodules. After more than 48 months of follow‐ups, the VRR of nodules was 96.9% (90.4%–100%). The symptoms and appearance were improved, with a postoperative recurrence rate of 1.35% (2/148) and an incidence of complications of 3.38%. Similar results were observed in our study. The VRR was 94% at 4 months after operation, and no complications occurred. This suggests that the MWA has similar efficacy and safety in both children and adults.

Ultrasound‐guided percutaneous MWA can be used for in situ ablation of thyroid nodules while maximizing the protection of surrounding normal thyroid tissue. The function of the thyroid gland is determined by thyroid hormone levels. In our study, the postoperative thyroid hormones were within the normal range, and there was no significant change in thyroid hormone levels between after surgery and before surgery, indicating that the MWA did not affect the thyroid function.

In summary, MWA is safe and effective in the treatment of benign thyroid nodules in children. This therapy is minimally invasive. It improves clinical symptoms and better protects the normal thyroid tissue, thereby minimizing the damage to thyroid function. This therapy has clinical application value and it is worthy of promotion. However, the following points need to be noted when performing MWA in children. Firstly, for the safety and effectiveness of the procedure, children have often to be treated with MWA under the general anesthesia. Therefore, more attention should be paid to protecting the recurrent laryngeal nerve during the surgery, especially in children with bilateral thyroid nodules. Besides, it is suggested that the intraoperative use of CEUS is helpful to quickly detect the existence of residual nodules, and timely supplementary ablation can minimize or avoid the recurrence caused by incomplete tumor ablation.^7^ Secondly, multidisciplinary communication may help to make a better treatment decision for children. Because MWA is more demanding in children than adults, it is necessary to carefully select appropriate patients for the MWA. In this way the children may benefit more. Thirdly, because the implementation of MWA is more challenging in children, it requires the doctor having better operating skills and more experienced in the procedure. Fourthly, because the neck tissues and organs are relatively loose, injection of separating liquid to form a separating zone is easy to be absorbed and diffused, and the liquid retention time is short. In order to avoid the thermal damage to the recurrent laryngeal nerve and blood vessels, the separating liquid can be injected multiple times in necessity according to the time of surgery so as to reduce the recurrent laryngeal nerve injury.^33^ There are limitations in the present study, including a small sample size, short follow‐up time, and single‐center study results. Therefore, large sample size and multicenter studies are still needed to further confirm the efficacy and safety of ultrasound‐guided MWA.

## AUTHOR CONTRIBUTIONS

Hongxia Zhang and Mingzhu Yu wrote the manuscript and collected data; Xiangru Kong initiated the idea, guided the article structure,and reviewed the final manuscript; Jingyu Chen,Caihui Hu and Yi Tang provided ultrasound follow‐up, contrast‐enhanced ultrasound and imaging guidance for this study; Zhenzhen Zhao and Chao Yang put forward constructive suggestions on literature retrieval; Yifei Du, Jian Sun, Jianwu Zhou and Xiaobin Deng completed some of the data analysis; All authors read and approved the final manuscript.

## CONFLICT OF INTEREST STATEMENT

The authors declare no conflict of interest.

## ETHICS STATEMENT

The authors declare that all methods were carried out in accordance with relevant guidelines and regulations. This study has been approved by the Ethics Committee of Children's hospital of Chongqing Medical University (Ethical Review for Research NO. 033; 2023). The informed consent of this study was obtained from all subjects' legal guardian(s).

## Data Availability

The data that support the findings of this study are openly available in 4TU. ResearchData at https://data.4tu.nl/private_datasets/X4EbNgRgjaSnZk5‐QLolk_FrNUWIcxdSDBxxOqumeIM.
